# Comparative Analysis of Single-Path and Multipath Adrenal Venous Sampling in Primary Aldosteronism

**DOI:** 10.1155/2023/8670365

**Published:** 2023-08-12

**Authors:** Zhoufei Fang, Han Cai, Qixiang Zhang, Jin Gong, Wei Zhou, Liangdi Xie, Feng Peng

**Affiliations:** ^1^Department of Geriatrics, The First Affiliated Hospital of Fujian Medical University, Fuzhou, China; ^2^Fujian Hypertension Research Institute, The First Affiliated Hospital of Fujian Medical University, Fuzhou, China; ^3^Clinical Research Center for Geriatric Hypertension Disease of Fujian Province, The First Affiliated Hospital of Fujian Medical University, Fuzhou, China; ^4^Branch of National Clinical Research Center for Aging and Medicine, The First Affiliated Hospital of Fujian Medical University, Fujian Province, Fuzhou, China; ^5^Department of Cardiology, National Regional Medical Center, Binhai Campus of the First Affiliated Hospital, Fujian Medical University, Fuzhou 350212, China; ^6^Department of Cardiology, The First Affiliated Hospital of Fujian Medical University, Fuzhou, China; ^7^The First Clinical Medical College of Fujian Medical University, Fuzhou, China

## Abstract

**Objective:**

To evaluate the safety and efficacy of adrenal venous sampling (AVS) via the cubital vein and femoral vein synchronously.

**Methods:**

A total of 200 patients with primary aldosteronism admitted to the First Hospital of Fujian Medical University were enrolled and randomly divided into a single-path AVS group (SP, *N* = 108) and a multipath AVS group (MP, *N* = 92). We analyzed the clinical characteristics, intubation success rate, procedure cost, total fluoroscopy time, complications, contrast dosage, and the number of catheters selected during AVS. A planar quadrant system was established to mark the direction of the adrenal opening, with the intersection of the right renal vein and the inferior vena cava defined as the origin. In digital subtraction angiography images, the RAV opening located in the 0–3 o'clock direction was the first quadrant (I), and the 3–6 o'clock direction was the third quadrant (III).

**Results:**

There was no statistical difference between the two groups at baseline. Multipath AVS had a significantly higher success rate of right-sided intubation than single-path AVS (success rate of right-sided intubation/%: SP 87.96 vs MP 95.65, *P* = 0.043). Total fluoroscopy time was significantly reduced (fluoroscopy time/min: SP 9.80 ± 4.07 vs MP 7.42 ± 3.48, *P* = 0.024) and the cost of the procedure was markedly lower (cost/yuan: SP 3,900.93 ± 1,191.12 vs MP 3,378.26 ± 399.40, *P* < 0.001). There was no significant difference in postoperative complications between the two groups. In the group I, the procedure was completed mainly with an MPA catheter (catheter selection/%: MPA 98.19 vs TIG 17.65, *P* < 0.001). In the group III, TIG catheters were used more frequently (catheter selection/%: MPA 1.81 vs TIG 82.35, *P* < 0.001).

**Conclusion:**

Multipath AVS via the cubital vein and femoral vein improves the success rate of AVS with comparable safety compared to single-path AVS. When the RAV is opened in the III quadrant, the TIG catheter improves the cannulation success rate. The multipath AVS method provides more catheter options. Patients diagnosed with PA at the First Hospital of Fujian Medical University from December 2019 to December 2021 were included. The collection of medical records of the included population was approved by the ethics committee (approval number: [2021] 311). This was a cross-sectional study in which some patients were treated surgically and some were treated with superselective adrenal artery embolization (SAAE). We conducted a cohort study of patients treated with SAAE. ClinicalTrials.gov Protocol Registration and Results System (PRS) receipt release date: January 11, 2022. This trial is registered with NCT05188872.

## 1. Introduction

Primary aldosteronism (PA) is a clinical syndrome characterized by high aldosterone, low renin activity, hypertension, and hypokalemia [1]. PA has different staging diagnoses. Adrenal venous sampling (AVS) is the basis for the staging diagnosis and determines the treatment strategy, which is the gold standard for determining PA staging and treatment strategy selection [2–4].

The high success rate of AVS has contributed to the development of unilateral adrenalectomy. Many studies have attempted to accomplish AVS through different vascular accesses, catheters, and devices.

When performing AVS, the catheter is superselectively embedded into the bilateral adrenal veins to obtain blood, and even when blood is obtained near the adrenal veins, the blood specimen will still be diluted, affecting the accuracy. Commonly selected sites for blood sampling are the elbow vein and the femoral vein [5]. The catheter is more easily accessible to the left adrenal vein (LAV), and the success rate of left-sided cannulation in technically mature AVS centers is above 95% [6].

Due to the different venous opening locations and imaging patterns, as well as the confusion of small branches of the surrounding hepatic veins, the success rate of the right adrenal gland is only about 80% [7].

In the past, researchers tried to improve the success rate of AVS and avoid adrenal vein hemodilution. Some studies proposed effective methods [8]. For example, Olivier et al. [9] applied cone beam CT before AVS, which could achieve precise intraoperative localization of the RAV. Maureen et al. [10] found that the catheter could be placed if a guiding vein was present during contrast. James et al. [11] used coaxial guidewire technique to suggest the success rate of cannulation.

To address the misclassification from asynchronous AVS of the right and left adrenal glands, some centers use continuous ACTH stimulation; however, ACTH stimulation may reverse the results of the lateralized side [12, 13]. Therefore, we need to improve the success rate of right-sided intubation and avoid the effects of asynchrony. On average, our center performs more than 100 AVS procedures per year.

This study is a single-center prospective study that showed an increased success rate of right-sided cannulation to 95%. The study found that the choice of catheter depended on the axial position of the central RAV. For example, when the RAV opening was in quadrant I, an MPA catheter may be the most appropriate. When the RAV opening was in quadrant III, the MPA catheter became difficult to detect. Using a catheter that fits the direction of the adrenal vein opening improved the success rate of AVS. The multipath method expanded catheter selection.

In addition, we used multipath AVS through the cubital vein and femoral vein to successfully complete simultaneous AVS. A comparison was made with single-path AVS to compare the effectiveness and safety of the new method. AVS is the gold standard for staging the diagnosis of PA, and we hope to obtain more information through the analysis.

## 2. Methods

### 2.1. Study Cohort (Figure 1)

The trial was a national, prospective, single-arm experimental pilot study. Blood screening (aldosterone-to-renin ratio, ARR) was performed at the First Hospital of Fujian Medical University from December 2019 to December 2021, followed by a confirmatory test (dynamic tests to suppress aldosterone production by salt loading, volume expansion, or inhibition of angiotensin-converting enzyme) from December 2019 to December 2021 in the First Affiliated Hospital of Fujian Medical University. A total of 200 patients were diagnosed with PA according to the criteria of an Endocrine Society Clinical Practice Guideline [1].

All patients underwent computed tomography (CT) and AVS and were randomized by a coin flip into two groups, including a single-path group and a multipath group. The single-path group used the cubital vein asynchronous AVS and the multipath group used synchronized AVS via the cubital vein and femoral vein.

This study was approved by the Ethics Committee of the First Affiliated Hospital of Fujian Medical University. The registration number was [2015]084 of the First Affiliated Hospital of Fujian Medical University.

### 2.2. Inclusion Criteria

**Figure 1:**
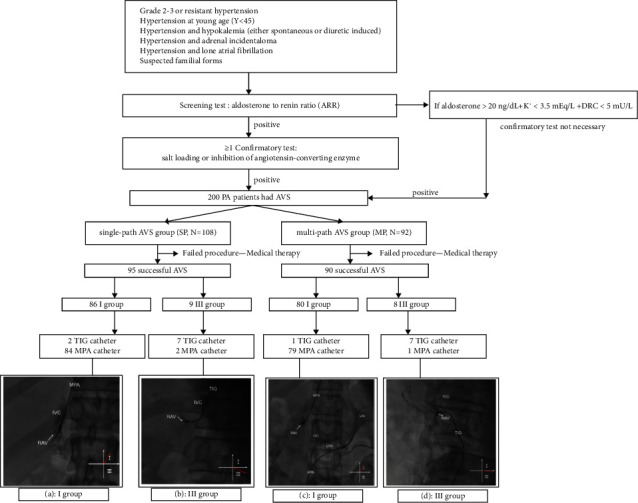
Flow diagram for diagnosis and grouping of PA patients. Defined the intersection of RAV and IVC as the origin and established a plane coordinate system. In digital subtraction angiography (DSA) images, the RAV opening located on 0–3 o'clock direction was the first quartile (I) and 3–6 o'clock direction was the third quadrant (III) according to clock wise notation. (a) DSA image showed a MPA catheter embedded in the RAV opening, with the adrenal vein opening towards the I quadrant. (b) DSA image showed the central RAV entering the IVC in the III quadrant, which was defined as group III. A TIG catheter embedded in the RAV. (c) DSA image showed 5F MPA catheter embedded in the RAV opening and in the LAV opening, respectively, and synchronously. (d) The RAV could not be reached by repeated use of MPA and TIG catheter through single-path AVS via the cubital vein. A TIG catheter was used to reach the RAV, which was shown to open in the III quadrant on angiography. PA: primary aldosteronism; AVS: adrenal venous sampling; DRC: direct renin concentration; ARR: aldosterone-to-renin ratio; RAV: right adrenal vein; LAV: left adrenal vein; IVC: inferior vena cava.

Patients with PA were established based on the standards and followed by the flowchart developed by the 2020 Working Group on Endocrine Hypertension of the European Society of Hypertension (Figure 1).

### 2.3. Exclusion Criteria

Patients who were with following conditions were excluded. (1) Patients with severe iodine allergy were at risk for contrast agent allergy. (2) Patients with abnormal coagulation must be corrected first. (3) Patients with acute stroke, acute myocardial infarction, and major surgical history within 1 month were excluded. (4) End-stage of the malignant tumor was excluded. (5) Patients with uncorrected hyperthyroidism were excluded for selected AVS. (6) There were no evidence-based medical studies to support pregnant women, lactation, and planned pregnancy patients undergo AVS. (7) Patients with severe systemic infection and being under mechanical ventilation were excluded.

### 2.4. Patients Demographic Characteristics

(1) Age, sex, height, weight, serum cholesterol, triglycerides, low-density lipoprotein cholesterol, creatinine, glomerular filtration rate, uric acid, and glycated hemoglobin were assessed at baseline. (2) Collected indicators reflecting PA characteristics, including systolic blood pressure (SBP), diastolic blood pressure (DBP), antihypertensive drugs, and biochemical indicators (plasma potassium, aldosterone, and renin).

Plasma renin, aldosterone, and aldosterone-renin ratio levels were assessed at baseline by a clinical laboratory (ACM Global Laboratories, Rochester, NY, USA). Patients abstained from all antihypertensive medications and were requested to fast prior to testing. Patients must be out of bed for at least 2 hours after resting and quietly sitting for a minimum of 5 minutes but preferably 30 minutes before blood was sampled.

The time and the patient's position (standing, sitting, or lying) at the time of blood collection were recorded. Later, the patient was asked to take the blood sample at the same time (±2 hours) and in the same position. (3) The primary efficacy of multipath AVS was evaluated by the success rate of unilateral intubation. Statistics of AVS intraoperative parameters were used to evaluate the minor efficacy of multipath AVS, including unilateral fluoroscopy time, total fluoroscopy time, contrast agent dosage, and operation cost. (4) The safety analysis between multipath AVS and single-path AVS included intraoperative complications (such as vascular dissection and vascular rupture) and postoperative complications (such as fever, hematoma, limb thrombosis, and death).

### 2.5. AVS Procedure

**Figure 2:**
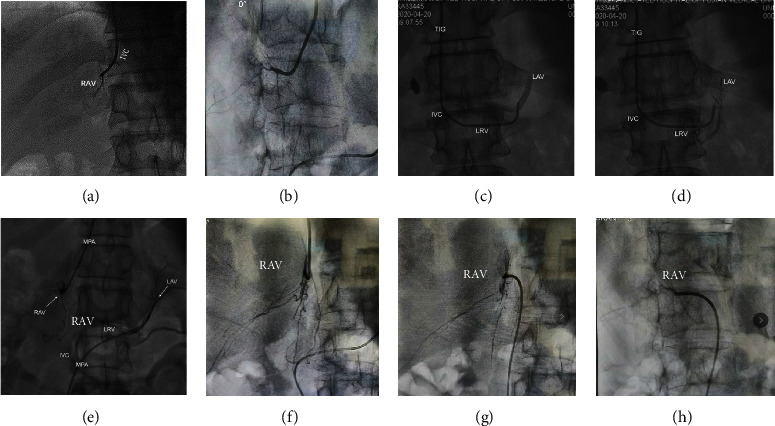
Techniques of using different catheters in single-path AVS and multipath AVS synchronously and asynchronously. (a) Inserted a 5F MPA catheter into the RAV in the single-path AVS group. (b) Inserted a 5F TIG catheter into the LAV in the single-path AVS group. Angiogram showed a thick common trunk vein formed by the confluence of the LAV and the PV. (c) To avoid local hemodilution of the LAV, a microcatheter was used to superselect into the LAV. Angiogram showed that the microcatheter superselected into the LAV. (d) 5F MPA catheter synchronously embedded in the RAV opening and in the LAV opening in the multipath AVS. (e) In anteroposterior position, intubated RAV through the cubital vein with a TIG catheter. (f) In right anterior oblique 30° angiography, the central venous opening of RAV was right oblique toward the III quadrant. (g) In anteroposterior position, intubated RAV with a MPA catheter via the femoral vein. (h) In right anterior oblique 30° angiography, the central venous opening of RAV was direct toward the III quadrant. Figures (e–h) show adrenal venography in the same patient in different positions. Figures (g, h) show that MPA catheter got through the femoral vein pathway and accurately positioned to the RAV with a total exposure time of 12.77 min. Figures (e, f) show that TIG catheter got through the cubital vein pathway, reaching the RAV with a total exposure time of 6.45 min. AVS: adrenal vein sampling; LAV: left adrenal vein; RAV: right adrenal vein; LRV: left renal vein; IVC: inferior vena cava; IEV: inferior emissary vein; PV: phrenic vein.

Single-path AVS was performed by a skilled operator. (1) After disinfection and anesthesia, the middle right cubital vein was punctured and a 5F sheath was inserted. (2) An X-ray was performed on the patient anterior chest and an F-type MPA catheter (Cordis, USA) was inserted into the inferior vena cava to collect a 2 ml adrenal vein blood sample. (3) A 5F MPA catheter was inserted into the RAV and a small amount of contrast medium was injected to determine the location. A 2 mL adrenal venous blood sample was taken (Figure 2(a)). If the 5F MPA multipurpose catheter cannot be inserted into the RAV, replace the TIG catheter (Figure 2(b)). (4) A TIG catheter (Cordis, USA) was inserted into the LAV, and a small amount of contrast medium was injected to determine the location. A 2 ml of venous blood sample was taken (Figure 2(c)).

Multipath AVS was performed by two skilled operators simultaneously. (1) The right cubital vein and right femoral vein were punctured after disinfection and anesthesia, and 5F sheaths were inserted, respectively. (2) An X-ray was performed on the patient anterior chest and an F-type MPA catheter (Cordis, USA) was inserted into the inferior vena cava to collect a 2 ml adrenal vein blood sample. (3) Operators simultaneously delivered the 5F MPA catheter (Cordis, USA) into bilateral adrenal veins, and then injected a small amount of contrast agent to determine the location (Figure 2(d)). If the 5F MPA catheter cannot be delivered into the RAV, the TIG catheter was replaced. (4) A 2 ml adrenal venous blood sample was taken simultaneously (Figure 2(e)).

### 2.6. AVS Parameter

Selectivity index (SI) was used to assess the adequacy of the adrenal vein cannulations and was calculated as Cortisol_adrenal wein_/Cortisol_periphrral wein_. Successful AVS was defined as SI > 2, bilaterally. Left/right fluoroscopy time was defined as the time for when the catheter entered the vascular sheath to the insertion of the catheter tip into the adrenal vein. Total fluoroscopy time was defined as from the start of the procedure to the end of hemostatic valve compression.

### 2.7. Statistical Analysis

All data were collected, statistically analyzed, and tabulated using the SPSS 22 software (SPSS Inc., Chicago, IL, USA). Continuous variables were expressed as mean ± SD, such as age, height, weight, SBP, DBP, cholesterol, triglycerides, low-density lipoprotein cholesterol, serum creatinine, uric acid, and glycated hemoglobin. Aldosterone, renin, and ARR were expressed as medians with interquartile ranges. Categorical data variables were shown as number (*n*) and percentage (%). For continuous data, the paired-samples *t*-test was used to compare the data between two groups. The chi-square test was used to compare the categorical data. All tests were two-tailed with 95% confidence intervals (CI), and *P* values <0.05 were considered statistically significant.

## 3. Results

### 3.1. Demographic Data (Table 1)

The SP-AVS group included 108 patients, 48 of whom were male, with a mean age of (51.07 ± 12.76) years. Baseline SBP was (157.15 ± 21.83) mmHg and baseline DBP was (92.16 ± 13.77) mmHg. In addition, 92 patients were included in the MP-AVS group with an age of (50.89 ± 12.77) years. Forty-five of them were male. Baseline SBP was (156.54 ± 21.54) mmHg and baseline DBP was (92.17 ± 12.81) mmHg. Serum potassium was (3.32 ± 0.56) mmol/L. There was no significant difference between the SP-AVS and MP-AVS groups in terms of baseline data.

### 3.2. AVS Parameters and Complications Analysis

Compared with the SP-AVS, the success rate of right-side intubation in the MP-AVS was higher (success rate of right-side intubation/%: SP-AVS 87.96% vs MP-AVS 95.65%, *P* = 0.043). Total fluoroscopy time was reduced (time/mins: SP-AVS 9.80 ± 4.07 vs MP-AVS 7.42 ± 3.48, *P* = 0.024). Also, the operation cost was lower in MP-AVS (cost/yuan: SP-AVS 3,900.93 ± 1,191.12 vs MP-AVS 3,378.26 ± 399.40, *P* < 0.001). There was no difference in postoperative complications between the two groups (Table 2).

### 3.3. Catheter Selection Analysis

In group I, MPA catheters were used in 163 cases (98.19%) and TIG catheters were used in 3 cases (1.81%). In group III, MPA catheters were used in 3 cases (17.65%) and TIG catheters were used in 14 cases (82.35%). As shown in Table 3, the frequency of using TIG catheters was significantly increased in group III compared with group I [*P*  <  0.001]. In LAV blood collection, 14% of the SP group used microcatheters and 2% of the MP group used microcatheters, which were significantly different [*P*  <  0.01].

## 4. Discussion

In this study, we perceptively explored the safety and efficacy of single-path AVS and multipath AVS via the femoral vein and cubital vein for primary aldosteronism. The findings were as follows: (1) Multipath AVS via the cubital vein and femoral vein significantly improved the success rate of AVS and had comparable safety compared to single-path AVS. (2) The TIG catheter improved the success rate when the RAV was opened towards the III quadrant. (3) The multipath AVS approach expanded catheter options. The results showed that the multipath AVS technique (simultaneous adrenal vein sampling through the calf vein and femoral vein) allowed simultaneous collection of bilateral adrenal vein blood samples and shortened the X-ray exposure time. Moreover, this method expanded the catheter selection without increasing the cost of the procedure or postoperative complications.

The multipath AVS technique was performed via the cubital vein and femoral vein synchronously. The modified multipath of AVS improved the success rate of AVS and enhanced the accuracy of diagnosis. AVS were initially performed using the SIMON catheter via the femoral vein single-path and later adopted single-path via the cubital vein by using a MPA catheter and a TIG catheter [14].

The disadvantages of single-path AVS were fewer catheter options and the inability to perform simultaneous bilateral adrenal venous blood collection. To address the problem of fluctuating cortisol secretion during AVS, asynchronous AVS was used in the early days, followed by stimulation of AVS with ACTH [15]. These methods reduce the reliability of the results. In addition, the success rate of AVS can be increased to 80–90% by cone beam CT, coaxial wire catheter technique, application of the inferior emissary vein, and precise manipulation by physicians [16].

However, in many studies, the prognosis of patients with AVS-guided PA was not superior to that of patients with CT-guided prognosis. Huang [17] and Rossi [18] suggested that AVS-guided PA treatment was more effective than CT-based treatment. Tanja [19] and Jessica [20] reported a negative result, which was not a problem with AVS guidance, but may be due to operational failure of AVS (failure rate of about 20%) and antilateral dominant secretion caused by ACTH stimulation. Therefore, it was essential to improve the success rate and simultaneous blood collection.

The AVS-guided prognostic analysis of PA is only of guiding value with accurate AVS results. While single-path AVS only allowed asynchronous extraction of adrenal blood, multipath AVS enabled synchronous blood extraction and allowed catheter selection based on the different opening positions of the adrenal veins in the planar quadrant. Only highly reliable AVS results were instructive for surgery. While single-path AVS only allowed asynchronous extraction of adrenal blood, multipath AVS enabled synchronous blood extraction and allowed catheter selection based on the different opening positions of the adrenal veins in the planar quadrant.

The right-sided adrenal had a variable opening, and route and catheter diversification improved the success of cannulation. When the left-sided adrenal veins opening was deep or overlapped with the phrenic vein, diversification avoided imprecise cannulation and hemodilution caused by the single use of TIG catheters. Multipath AVS via the cubital vein and femoral vein significantly improved the success rate of AVS. ACTH stimulation was not required, which can reflect the results of lateral secretion. Also, incorrect typing diagnosis due to fluctuations in aldosterone and cortisol secretion in asynchronous AVS can be avoided.

There were no reports on the use of TIG catheters in the AVS to access the RAV. We defined the axial location of the RAV opening and found a significant increase in the use of TIG catheters in the III quadrant. Multipath AVS expanded the options for alternative catheters, especially when MPA catheters cannot be inserted into the adrenal vein in conventional AVS. Due to the multipath AVS approach, alternative catheters such as TIG, SIMON, or VER 135° catheters can be used when the MPA catheter cannot be inserted into the adrenal vein.

The TIG catheter improved the success of the operation because the TIG catheter had the advantage of bending upward proximally, and the second curvature of the catheter provided support when the TIG catheter was formed in the inferior vena cava. Therefore, we found that for patients with RAV opening in the III quadrant, the TIG catheter was more suitable for RAV implantation via the cubital vein.

Our previous studies demonstrated the safety and feasibility of multipath AVS [21]. Synchronous AVS via the cubital vein and femoral vein improved the success rate of bilateral intubation and reduce the fluoroscopy time and operation cost, and the simultaneous operation by both operators reduced the difficulty of the procedure. We also analyzed the relationship between the axial position of the RAV opening in AVS and catheter selection.

We observed an interesting phenomenon. After the TIG entered the RAV via the cubital vein, we attempted a right anterior oblique 30° angiogram as the RAV almost overlapped the inferior vena cava on anterior-posterior fluoroscopy (Figure 2(e)) and found that the RAV opened downward as estimated (Figure 2(g)). A MPA catheter was used to reach the RAV via the femoral vein, which confirmed the location of the RAV in the third quadrant and the feasibility of using TIG. DSA image confirmed the initial assessment (Figure 2(h)). This would facilitate the AVS procedure when multipath was available in both the cubital vein and the femoral vein.

In single-path AVS, the RAV is reached using an MPA catheter, and a TIG catheter was the only option for hooking the LAV opening. Sometimes only the first branch of the adrenal vein can be hooked through the cubital vein. For deeper openings in the left adrenal vein or openings overlapped with the phrenic vein, a microcatheter was required. The above were the reasons why single-path AVS was costlier. The variety of paths and catheters avoided imprecision of cannulation and hemodilution. Multipath access expanded the choice of catheters. The lower cost of the procedure and fewer postoperative complications demonstrated the superiority of multipath AVS.

## 5. Conclusion

This is the first study on the operational innovation of AVS that demonstrates the effectiveness and safety of simultaneous AVS treatment through the cubital vein and femoral vein, expanding the choice of catheters. The TIG catheter via the femoral vein is an option when the RAV is open towards the third quadrant.

Single-center clinical studies, small samples, and existing selection bias are limitations of this study. This report is a preliminary study, and as the number of patients increases, we will conduct a multicenter, large-sample clinical study. In the future, we will include multiple catheters in the trial, such as the SIMON catheter and the VER135° catheter. We hope to increase the success rate of AVS to 100% and provide the gold standard of diagnosis for the treatment of primary aldosteronism.

## Figures and Tables

**Table 1:** Characteristics of baseline data between single-path group and multipath group with primary aldosteronism patients receiving AVS.

	Single-path AVS (SP, no. = 108)	Multipath AVS (MP, no. = 92)	*P* value (SP vs MP)
Male	48 (44.44%)	45 (48.91%)	0.57
Age (years)	51.07 ± 12.76	50.89 ± 12.77	0.82
Height (cm)	165.96 ± 8.37	164.92 ± 8.41	0.98
Weight (kg)	68.65 ± 11.27	67.14 ± 10.61	0.51
SBP (mmHg)	157.15 ± 21.83	156.54 ± 21.54	0.98
DBP (mmHg)	92.16 ± 13.77	92.17 ± 12.81	0.42
Antihypertensive drugs	2 (1, 2)	2 (1, 2)	0.47
Cholesterol (mmol)	4.10 ± 0.80	4.10 ± 0.81	0.86
Triglyceride (mmol)	1.48 ± 0.94	1.48 ± 0.90	0.79
LDL (mmol)	2.62 ± 0.80	2.60 ± 0.78	0.82
Creatinine (*μ*mol/L)	74.52 ± 48.81	74.45 ± 52.27	0.87
Uric acid(mmol)	358.01 ± 104.27	353.14 ± 103.85	0.91
Glycosylated hemoglobin (%)	5.63 ± 0.86	5.64 ± 0.91	0.90
Serum potassium(mmol)	3.33 ± 0.58	3.32 ± 0.56	0.87
Supine ALD (ng/dl)	20.57 (14.97, 27.40)	21.14 (15.18, 25.69)	0.89
Supine DRC (*μ*IU/ml)	0.50 (0.20, 0.70)	0.50 (0.20, 0.62)	0.88
Supine ARR (ng/dl)	55.58 (30.54, 81.41)	56.50 (35.30, 100.00)	0.93
Upright ALD (ng/dl)	21.52 (16.29, 28.34)	21.67 (16.29, 29.12)	0.94
Uprigh DRC (*μ*IU/ml)	0.55 (0.24, 1.06)	0.50 (0.20, 0.95)	0.82
Upright ARR (ng/dl)	35.12 (20.99, 75.59)	50.23 (26.85, 82.01)	0.48
Aldosteronoma	42 (38.89%)	38 (43.48%)	0.57
Adrenal hyperplasia	66 (61.11%)	52 (56.52%)	0.57

SBP: systolic blood pressure; DBP: diastolic blood pressure; LDL: low-density lipoprotein cholesterol; ALD: serum aldosterone; DRC: serum renin concentration; ARR: aldosterone-to-renin ratio. Age was calculated in years from birthdate to date of informed consent.

**Table 2:** Parameters and complications of AVS.

	Single-path AVS (SP, no. = 108)	Multipath AVS (MP, no. = 92)	*P* value (SP vs MP)
Success rate of right-side intubation	95 (87.96%)	88 (95.65%)	0.04^★^
Success rate of left-side intubation	105 (97.22%)	90 (97.83%)	1.00
Right fluoroscopy time (min)	5.58 ± 3.37	5.28 ± 4.09	0.48
Left fluoroscopy time (min)	3.27 ± 1.67	3.27 ± 1.72	0.67
Total fluoroscopy time (min)	9.80 ± 4.07	7.42 ± 3.48	0.02^*∗*^
Dosage of contrast (ml)	30.55 ± 7.35	30.04 ± 8.07	0.46
Operation cost (yuan, RMB)	3,900.93 ± 1,191.12	3,378.26 ± 399.40	<0.001^*∗*^
Intraoperative complications			
Vascular dissection	2 (1.90%)	—	0.50
Vascular rupture	—	—	—
Postoperative complications			
Fever	1 (0.93%)	1 (0.11%)	1.00
Hematoma	—	2 (2.20%)	0.21
Limb thrombosis	—	—	—
Death	—	—	—

Success rate of right- or left-side intubation, vascular dissection, fever, and hematoma using correction *χ*^2^ test. ^★^*P* < 0.05 vs SP-AVS group by correction *χ*^2^ tests. ^*∗*^*P* < 0.05 vs SP-AVS group by paired-samples *t*-tests.

**Table 3:** Relationship of catheter selection and right adrenal vein opening axial position.

	I group	III group	Total
MPA catheter	163 (98.19%)	3 (1.81%)	166
TIG catheter	3 (17.65%)	14 (82.35%)^★^	17
Total	166	17	183

^★^ *P* < 0.05 vs I group by correction *χ*^2^ tests.

## Data Availability

The raw data supporting the conclusions of this article will be made available by the authors without undue reservation.
